# The Impact of Non‐Radical Hysterectomy on Urinary Functions: Evaluation of Symptoms—A Systematic Review and Meta‐Analysis

**DOI:** 10.1111/1471-0528.70056

**Published:** 2025-10-17

**Authors:** Roberta Maria Arseni, Emanuele De Angelis, Ilaria Cuccu, Andrea Giannini, Giorgio Bogani, Francesco Plotti, Corrado Terranova, Roberto Angioli, Tommaso Simoncini, Giuseppe Vizzielli, Stefano Restaino, Jvan Casarin, Massimiliano Fambrini, Flavia Sorbi, Paolo Scollo, Fulvio Zullo, Eliona Demaliaj, Giorgia Perniola, Ludovico Muzii, Violante Di Donato

**Affiliations:** ^1^ Department of Maternal and Child Health and Urological Sciences Sapienza University of Rome, Policlinico Umberto I Rome Italy; ^2^ Unit of Gynecology, Department of Surgical and Medical Sciences and Translational Medicine Sant'Andrea Hospital, Sapienza University of Rome Rome Italy; ^3^ Gynecological Oncology Unit Fondazione IRCCS Istituto Nazionale dei Tumori Milan Italy; ^4^ Research Unit of Gynaecology, Department of Medicine and Surgery Università Campus Bio‐Medico di Roma Rome Italy; ^5^ Division of Obstetrics and Gynecology I, Department of Clinical and Experimental Medicine University of Pisa Pisa Italy; ^6^ Clinic of Obstetrics and Gynecology, Santa Maria della Misericordia University Hospital Azienda Sanitaria Universitaria Friuli Centrale Udine Italy; ^7^ Obstetrics and Gynecology Department University of Insubria Varese Italy; ^8^ Division of Obstetrics and Gynecology, Department of Experimental and Clinical Biomedical Sciences “Mario Serio” Careggi University Hospital Florence Italy; ^9^ Department of Medicine and Surgery University of Enna “Kore” Enna Italy; ^10^ Department of Obstetrics and Gynecology University of Catanzaro Catanzaro Italy; ^11^ Department of Obstetrics and Gynecology University Hospital “Queen Geraldine” Tirane Albania

**Keywords:** non‐radical hysterectomy, stress incontinence, urge incontinence, urinary incontinence, urodynamic disfunction, urodynamic tests

## Abstract

**Background:**

Simple hysterectomy is one of the most common gynaecological surgical procedures worldwide; however, its association as a possible aetiological factor for urinary dysfunction remains controversial.

**Objective:**

To evaluate the clinical impact of different types of non‐radical hysterectomy on lower urinary tract symptoms (LUTS).

**Search Strategy:**

A structured search was conducted across scientific sources through December 1989 to March 2025, using terms including: ‘urodynamics’, ‘urinary incontinence’, ‘stress incontinence’, ‘urge incontinence’, ‘urinary urgency’, ‘urinary frequency’, ‘urinary nocturia’ and ‘urinary retention’, ‘lower urinary tract symptoms’, ‘hysterectomy’.

**Selection Criteria:**

Randomised controlled trials and prospective observational studies assessing patients undergoing simple hysterectomy with pre‐ and post‐operative evaluation by validated questionnaires. Exclusion criteria included case reports, reviews, editorials, short communications, radical hysterectomy, post‐operative assessment only, non‐English publications and studies on pelvic organ prolapse surgery.

**Data Collection and Analysis:**

Ten studies, encompassing 1769 patients, were included in the analysis. Five outcomes were selected: changes in urinary frequency; occurrence of stress urinary incontinence; occurrence of urge urinary incontinence; changes in nocturia; changes in incomplete bladder emptying.

**Main Results:**

Changes from baseline to last follow‐up available in urinary frequency (OR 0.48, 95% CI 0.36–0.66; *p* < 0.00001); stress urinary incontinence (OR = 0.54, 95% CI 0.44–0.68; *p* < 0.00001); urge urinary incontinence (OR = 0.76, 95% CI 0.72–0.94; *p* = 0.01); nocturia (OR 0.55, 95% CI 0.36–0.84; *p* = 0.005); incomplete bladder (OR = 0.95, 95% CI 0.66–1.36; *p* = 0.77).

**Conclusion:**

The present meta‐analysis suggests that simple hysterectomy is associated with a reduction in the prevalence of urinary symptoms postoperatively.

**Trial Registration:**

PROSPERO: CRD42024575574

## 1 Introduction

Hysterectomy is one of the most common gynaecological surgical procedures worldwide [1], and it is often performed for benign gynaecological conditions such as uterine leiomyomas and dysfunctional uterine bleeding [2]. Hysterectomy can be performed via different surgical approaches including vaginal, open, laparoscopic or robotic‐assisted [3]. The type of surgical approach depends on clinical conditions, such as the size of the uterus, the patient's comorbidities and preferences and the surgeon's skills [4].

Several studies have described post‐hysterectomy sequelae [5], yet the literature is heterogeneous and controversial with discordant incidences, both due to the lack of control groups and to variability in the reference populations and indications for the procedure.

Among the urological sequelae and pelvic floor symptoms, hysterectomy has been recognised as one of the potential aetiological factors contributing to urinary dysfunction [6].

Lower urinary tract symptoms (LUTS), particularly urinary incontinence (UI), are one of the postoperative sequelae of hysterectomy [7]. UI is defined as the involuntary leakage of urine from the urethra that occurs when intravesical pressure exceeds urethral closing pressure. In clinical practice, a distinction can be made between stress urinary incontinence (SUI) and urge urinary incontinence (UUI). SUI involves the involuntary loss of urine with effort or exercise, such as sneezing or coughing. UUI is characterised by the involuntary loss of urine accompanied by or immediately preceded by a strong, unexpected and uncontrollable urge to void due to involuntary contractions of the detrusor muscle [8].

The gold standard for objectively diagnosing urinary incontinence is through urodynamic studies, such as cystometry and uroflowmetry. However, subjective information obtained from validated questionnaires and interviews is also valuable [9].

Since it is well established that radical hysterectomy damages the pelvic autonomic nerves, leading to long‐term urinary and sexual dysfunction [10, 11, 12], studies on simple total (non‐radical) hysterectomy for benign conditions are controversial; some report a worsening of symptoms after hysterectomy [13, 14], while others report beneficial effects [15, 16, 17]. Nevertheless, this variation may partly result from preoperative symptoms. Many women undergoing hysterectomy for benign conditions already present with urinary symptoms; therefore, not all postoperative urinary issues can be attributed solely to the gynaecological surgery. Consequently, to detect the effects of hysterectomy on lower urinary functions, it is necessary to report pre‐ and postoperative urinary assessment. The aim of the present meta‐analysis is to analyse the clinical impact of different types of non‐radical hysterectomy on urologic symptoms and urodynamic dysfunction.

## 2 Materials and Methods

### 2.1 Search Strategy

In April 2025, a comprehensive literature review was conducted, covering studies from database inception through March 2025. The following databases were systematically searched: PubMed/MEDLINE, Embase and Cochrane Central Register of Controlled Trials (CENTRAL). A predefined Boolean search strategy was applied.

The search included the following terms and their MeSH equivalents: ‘urodynamics’, ‘urinary incontinence’, ‘stress incontinence’, ‘urge incontinence’, ‘urinary urgency’, ‘urinary frequency’, ‘urinary nocturia’, ‘urinary retention’, ‘lower urinary tract symptoms’ ‘hysterectomy’.

Additional manual screening of reference lists from relevant literature was conducted.

Article abstracts and, where appropriate, the full text of articles and cross‐referenced studies identified from retrieved articles were screened for pertinent information. All duplicate records were removed. The overall search strategy was carried out following PRISMA (Preferred Reporting Items for Systematic Reviews and Meta‐Analyses) guidelines.

### 2.2 Study Selection and Methodologic Quality Assessment

The studies were selected independently by three authors (R.A., E.D.A., I.C.). Publications were evaluated based on predefined inclusion and exclusion criteria. Inclusion criteria were as follows: (1) randomised controlled and prospective observational studies; (2) patients undergoing total or subtotal hysterectomy; (3) patients assessed by urodynamic studies or validated questionnaires both pre‐ and post‐operatively. The following exclusion criteria were adopted: (1) case reports, editorials, reviews and short communications; (2) patients undergoing radical hysterectomy; (3) patients assessed by urodynamic studies or validated questionnaires only post‐operatively; (4) publications in a non‐English language; (5) pelvic organ prolapse surgery.

Data extraction from each included study was based on study characteristics and predefined outcome variables. The following variables were retrieved: year of publication, country, study design and setting, sample size, mean age, body mass index (BMI) where available, ethnicity, surgical indication, type of hysterectomy (total, subtotal, laparoscopic, vaginal, robotic), follow‐up duration and type of urodynamic studies or validated questionnaires utilised.

The methodological quality assessment was performed following the Cochrane Handbook for Systematic Reviews of Interventions v.5.1.0.

### 2.3 Outcomes

Five groups of outcomes that are particularly concerning for patients in this context were evaluated:

- Changes in urinary frequency after hysterectomy: Changes from baseline to 3, 6 and 12 months or at last follow‐up available have been extrapolated from the studies; stratifying according to the different surgical techniques used.
- Occurrence of stress urinary incontinence after hysterectomy: changes from baseline to 3, 6 and 12 months or at last follow‐up available have been extrapolated from the studies; stratifying according to different surgical techniques used.
- Occurrence of urge urinary incontinence after hysterectomy: changes from baseline to 3, 6 and 12 months, or at last follow‐up available, have been extrapolated from the studies; stratifying according to the different surgical techniques used.
- Changes in nocturia after hysterectomy: changes from baseline to 3, 6 and 12 months or at last follow‐up available have been extrapolated from the studies; stratifying according to the different surgical techniques used.
- Changes in incomplete bladder emptying after hysterectomy; changes from baseline to 3, 6 and 12 months or at last follow‐up available have been extrapolated from the studies; stratifying according to different surgical techniques used.

### 2.4 Statistical Analysis

A meta‐analysis of aggregate data was conducted to generate a pooled estimate using effect estimates of individual studies reported in the published literature. The data were analysed using RevMan software (Review Manager version 5.4, the Cochrane Collaboration). Dichotomous outcomes from each study were represented as odds ratio (OR) with 95% confidence interval (CI). Continuous outcomes were represented as mean differences (MD). A *p*‐value of < 0.05 was considered statistically significant. Heterogeneity among studies was reported with the *I* ^2^ statistic. A random‐effects model was applied in the meta‐analysis if heterogeneity was detected, while a fixed‐effects model was used if heterogeneity was absent. We adopt to assess publication bias using Egger's test and funnel plots if the number of studies was 10 or more, as these analyses are otherwise underpowered.

## 3 Results

### 3.1 Study and Participants' Characteristics

The systematic search yielded 37 relevant studies (Figure S1). Of these, 27 were excluded because they did not have validated questionnaires both pre‐ and post‐operatively or for using a surgical technique to repair the prolapse. Ten studies [13, 15, 16, 17, 18, 19, 20, 21, 22, 23] fulfilled the predefined inclusion criteria (Table S1).

Across the 10 included studies, the mean age of participants ranged from 43 to 48 years. Body mass index (BMI) and ethnicity were reported in only a single Finnish cohort [22] which included 14% of overweight patients, thereby limiting the generalisability of these findings. Indications for hysterectomy were benign conditions, most frequently leiomyomas and abnormal uterine bleeding, adenomyosis or other benign gynaecological condition.

### 3.2 Risk of Bias

The Risk of Bias in Non‐Randomized Studies of Interventions (ROBINS‐I) tool was used to assess the risk of bias in non‐randomised studies of interventions included in the meta‐analysis as detailed in Table S2.

### 3.3 Effects of Interventions

#### 3.3.1 Changes in Urinary Frequency

**FIGURE 1:**
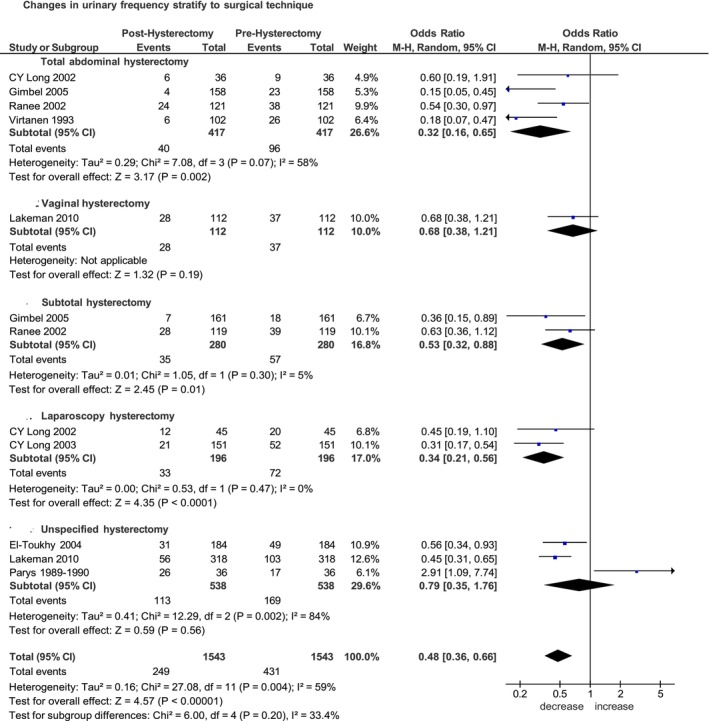
Forest plot: Changes in the incidence of urinary frequency before and after hysterectomy, stratified by surgical technique.

Of the eight selected studies published between 1989 and 2025 [13, 15, 16, 18, 19, 20, 22, 23], five were prospective non‐randomised studies [13, 16, 20, 22, 23] and three were randomised controlled trials [15, 18, 19]. The median follow‐up time across the studies ranged from 6 weeks to 3 years. The increase in frequency was evaluated with a cut‐off greater than six times per day in three studies [13, 15, 23], greater than seven times in two studies [16, 18], greater than or equal to eight times in one study [22], greater than 10 times in one study [19] and with the urogenital distress inventory (UDI) in one study [20]. Of the 1543 women included in the pooled analysis, a frequency increase was reported in 249 (16.1%) women post‐hysterectomy compared to 431 (27.9%) before hysterectomy. Overall, the increase in urinary frequency was significantly reduced after hysterectomy. OR of 0.48 (95% CI 0.36–0.66; *p* < 0.00001) (Figure 1).

In the stratified analysis by surgical technique, total abdominal hysterectomy was associated with a significant postoperative reduction in urinaryfrequency among patients undergoing open hysterectomy. Specifically, four studies including 417 women undergoing total abdominal hysterectomy (TAH) [15, 18, 19, 22] yielded a pooled OR of 0.39 (95% CI 0.17–0.86; *p* = 0.02), and two studies including 280 women undergoing subtotal hysterectomy (STH) [18, 19] yielded a pooled OR of 0.32 (95% CI 0.16–0.65; *p* = 0.002) (Figure 1).

In the subgroup of total hysterectomy, a significant postoperative reduction in urinary frequency was observed after both open [15, 18, 19, 22] (OR 0.32, 95% CI 0.16–0.65; *p* = 0.002) and laparoscopic hysterectomy [15, 23] (OR 0.34, 95% CI 0.21–0.56; *p* < 0.0001). For vaginal hysterectomy [20], only one study was available, which made a pooled subgroup analysis impracticable (Figure S2).

Stratifying according to follow‐up time: respectively, six studies of 1110 women after 6 months [13, 16, 18, 20, 22, 23] and three studies involving 661 women after 1 year [18, 19, 22] showing a significant reduction during this follow‐up. The pooled estimated odds ratios were 0.53 (95% CI 0.34–0.83; *p* = 0.006) and 0.32 (95% CI 0.15–0.68; *p* = 0.003), respectively (Figure S3).

#### 3.3.2 Changes in Rate of Stress Urinary Incontinence After Hysterectomy

Of nine selective studies were published between 1989 and 2025 [13, 15, 16, 17, 19, 20, 21, 22, 23], six were prospective non‐randomised studies [13, 16, 17, 20, 22, 23] and three randomised controlled trials [15, 19, 21]. The median follow‐up time ranged from 6 weeks to 3 years. Of the 1491 women included in the pooled analysis, the symptoms of SUI occurred in 314 (21.2%) women post‐hysterectomy compared to the 481 (32.3%) women before hysterectomy.

**FIGURE 2:**
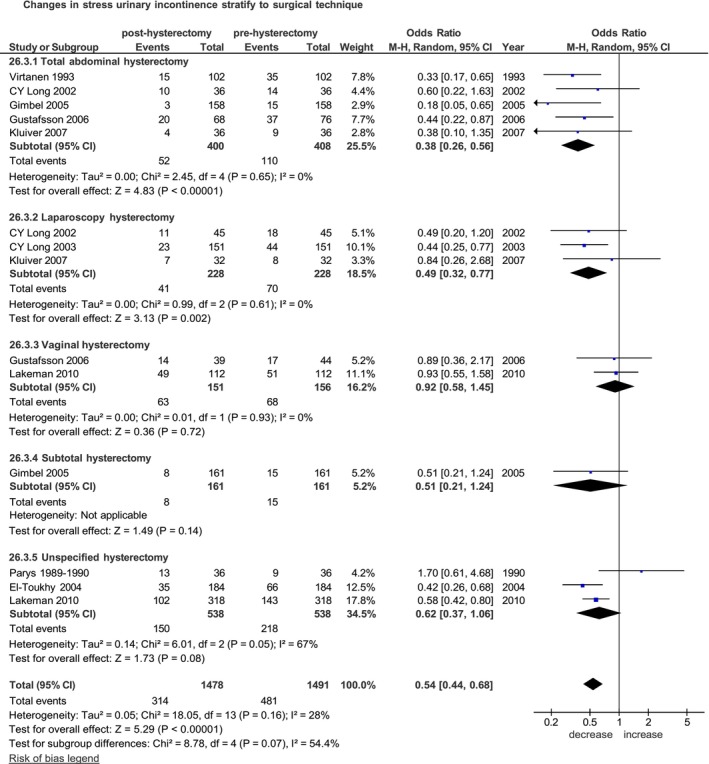
Forest plot: Changes in the incidence of stress urinary incontinence before and after hysterectomy, stratified by surgical technique.

Overall, the incidence of SUI was significantly lower after hysterectomy (OR = 0.54, 95% CI 0.44–0.68; *p* < 0.00001) (Figure 2).

Stratified analysis according to surgical technique, in the subgroups of open hysterectomy, total abdominal hysterectomy was associated with a significant postoperative reduction in SUI. Specifically, five studies including 408 women undergoing TAH [15, 17, 19, 21, 22] yielded a pooled OR of 0.38 (95% CI 0.26–0.56; *p* < 0.00001), and one study including 161 women undergoing STH [19] yielded a pooled OR of 0.51 (95% CI 0.21–1.24; *p* = 0.14) (Figure 2).

In the subgroup of total hysterectomy, the comparison across surgical routes demonstrated a significant postoperative reduction in SUI after both TAH and LTH. The effect was consistent for open total hysterectomy [15, 17, 19, 21, 22] (OR 0.38, 95% CI 0.26–0.56; *p* < 0.00001) and laparoscopic hysterectomy [15, 21, 23] (OR 0.49, 95% CI 0.32–0.77; *p* = 0.002), whereas no significant reduction was observed for vaginal hysterectomy [17, 20] (OR 0.92, 95% CI 0.58–1.45; *p* = 0.72) (Figure S4).

Stratifying according to follow‐up time: respectively, five studies of 870 women after 6 months [13, 16, 20, 22, 23], three studies of 541 women after 1 year [17, 19, 22] and two studies of 440 women after 3 years [17, 20]. The pooled ORs 0.50 (95% CI 0.35–0.69; *p* < 0.0001), 0.48 (95% CI 0.34–0.68; *p* < 0.0001) and 0.79 (95% CI 0.61–1.02; *p* = 0.07), respectively (Figure S5).

#### 3.3.3 Changes in Urge Urinary Incontinence Rate After Hysterectomy

Nine of the selected studies were published between 1993 and 2025 [15, 16, 17, 18, 19, 20, 21, 22, 23]. Five were prospective non‐randomised studies [16, 17, 20, 22, 23] and four were randomised controlled trials [15, 18, 19, 21]. The median follow‐up time across the studies ranged from 6 weeks to 3 years. Of the 1692 women included in the pooled analysis, the symptoms of UUI were reported in 181 (10.8%) women post‐hysterectomy and in 230 (13.6%) women before hysterectomy.

**FIGURE 3:**
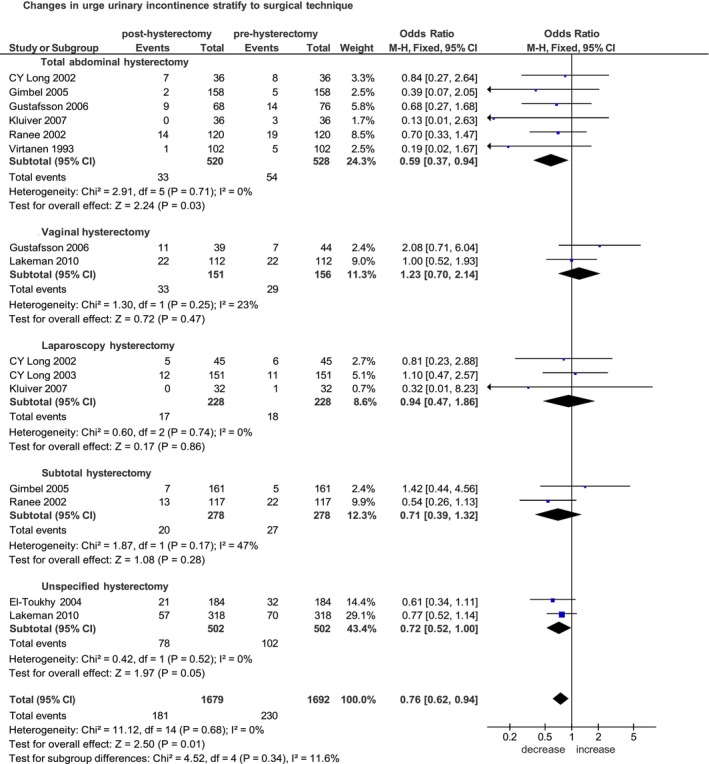
Forest plot: Changes in the incidence of urge urinary incontinence before and after hysterectomy, stratified by surgical technique.

Overall, UUI symptoms significantly decrease after hysterectomy (OR 0.76, 95% CI 0.62–0.94; *p* = 0.01) (Figure 3).

Stratified analysis according to surgical technique, in the subgroups of open hysterectomy, total abdominal hysterectomy was associated with a significant postoperative reduction in UUI. Specifically, six studies including 528 women undergoing TAH [15, 17, 18, 19, 21, 22] yielded a pooled OR of 0.59 (95% CI 0.37–0.94; *p* = 0.03) and two studies including 278 women undergoing STH [17, 20] yielded a pooled OR of 0.71 (95% CI 0.39–1.32; *p* = 0.28) (Figure 3).

In the subgroup of total hysterectomy, the comparison across surgical routes also demonstrated a significant postoperative reduction in UUI in the group of TAH. The effect was consistent for open total hysterectomy [15, 17, 18, 19, 21, 22] (OR 0.59, 95% CI 0.37–0.94; *p* = 0.03), whereas no significant reduction was observed for vaginal hysterectomy [17, 20] (OR 1.23, 95% CI 0.70–2.14; *p* = 0.47) and laparoscopic hysterectomy [18, 19, 23] (OR 0.94, 95% CI 0.47–1.86; *p* = 0.86) (Figure S6).

Stratifying according to follow‐up time: respectively, five studies of 1104 women after 6 months [16, 18, 20, 22, 23], four studies of 778 women after 1 year [17, 18, 19, 22] and two studies of 440 women after 3 years [17, 20]. The pooled ORs were 0.62 (95% CI 0.48–0.79; *p =* 0.0001), 0.70 (95% CI 0.48–1.01; *p =* 0.06) and 1.13 (95% CI 0.83–1.53; *p* = 0.43), respectively (Figure S7).

#### 3.3.4 Changes in Nocturia After Hysterectomy

**FIGURE 4:**
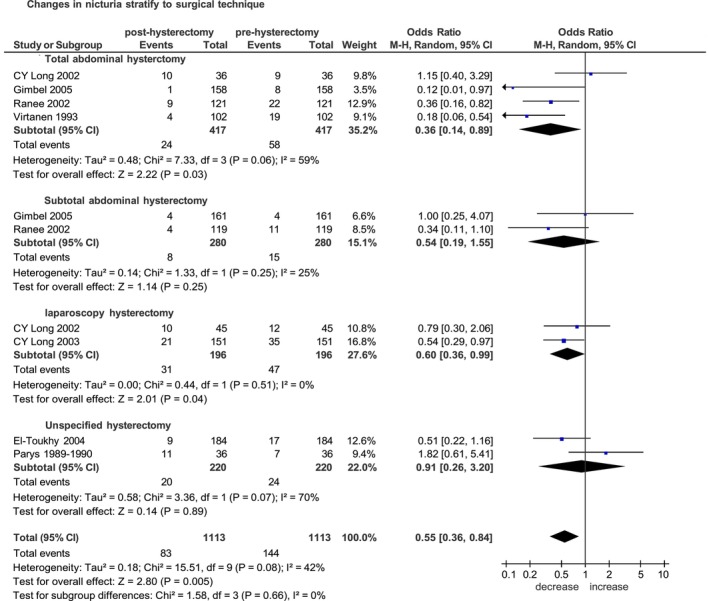
Forest plot: Changes in the incidence of nicturia before and after hysterectomy, stratified by surgical technique.

Of the seven selected studies published between 1989 and 2025 [13, 15, 16, 18, 19, 22, 23], four were prospective non‐randomised studies [13, 16, 22, 23] and three were randomised controlled trials [15, 18, 19]. The median follow‐up time across the studies varied from 6 weeks to 1 year. The increase in nocturia was evaluated with a cut‐off of more than one time per day in five studies [13, 16, 19, 22, 23] and greater than two times in two studies [15, 18]. Of the 1113 women included in the pooled analysis, the increase in nocturia was reported in 83 (7.5%) women post‐hysterectomy and in 144 (12.9%) women before hysterectomy. The overall increase in nocturia was reduced after hysterectomy (OR 0.55, 95% CI 0.36–0.84; *p* = 0.005) (Figure 4).

Stratified analysis according to surgical technique, in the subgroups of open hysterectomy, total abdominal hysterectomy was associated with a significant postoperative reduction in nocturia. Specifically, four studies including 417 women undergoing TAH [15, 18, 19, 22] yielded a pooled OR of 0.36 (95% CI 0.14–0.89; *p* = 0.03) and two studies including 280 women undergoing STH [18, 19] yielded a pooled OR of 0.54 (95% CI 0.19–1.55; *p* = 0.25) (Figure 4).

In the subgroup of total hysterectomy, the comparison across surgical routes also demonstrated a significant postoperative reduction in nocturia after both TAH and LTH. The effect was consistent for open total hysterectomy [15, 18, 19, 22] (OR 0.36, 95% CI 0.14–0.89; *p* = 0.03) and laparoscopic hysterectomy [15, 22] (OR 0.60, 95% CI 0.36–0.99; *p* = 0.04) (Figure S8).

Stratifying according to time of follow‐up: five studies of 713 women after 6 months [13, 16, 18, 22, 23] and three studies of 661 women after 1 year [18, 19, 22]. The pooled ORs were 0.51 (95% CI 0.37–0.71; *p* < 0.0001) and 0.44 (95% CI 0.26–0.75; *p* = 0.002), respectively (Figure S9).

#### 3.3.5 Changes in Incomplete Bladder Emptying After Hysterectomy

Of the seven selected studies published between 1989 and 2025 [13, 15, 16, 18, 19, 22, 23], four were prospective non‐randomised studies [13, 16, 22, 23] and three were randomised controlled trials [15, 18, 19]. The median follow‐up time across the studies ranged from 6 weeks to 1 year. Of the 1111 women included in the pooled analysis, incomplete bladder emptying occurred in 188 (16.9%) women post‐hysterectomy and in 202 (18.2%) women before hysterectomy (OR 0.95, 95% CI 0.66–1.36; *p* = 0.77) (Figure S10).

Stratified analysis according to surgical technique demonstrated no significant postoperative reduction in incomplete bladder emptying in both subgroups of open hysterectomy (Figure S10).

In the subgroup of total hysterectomy, the comparison across surgical routes also demonstrated no significant postoperative reduction in incomplete bladder emptying in all subgroups. The effect was consistent for open total hysterectomy [15, 18, 19, 22] (OR 0.68, 95% CI 0.38–1.23; *p* = 0.20) and laparoscopic hysterectomy [15, 23] (OR 0.81, 95% CI 0.48–1.36; *p* = 0.43) (Figure S11).

Stratifying according to follow‐up time: four studies of 560 women after 6 months [13, 16, 18, 22] and three studies of 659 women after 1 year [18, 19, 22]. ORs were 0.76 (95% CI 0.40–1.47; *p* = 0.42) and 0.54 (95% CI 0.39–0.73; *p* = 0.001), respectively (Figure S12).

## 4 Discussion

### 4.1 Main Findings

The present meta‐analysis demonstrates a decrease in urinary symptoms after simple hysterectomy, based on last follow‐up from baseline, regardless of the surgical technique performed. In particular, it shows a significant reduction in urinary frequency (OR 0.48; *p* < 0.00001), SUI (OR 0.54; *p* < 0.00001), UUI (OR 0.76; *p* = 0.01) and nocturia (OR 0.55; *p* = 0.005). These findings highlight the potential benefits of simple hysterectomy on urinary function, providing clinically relevant insights.

First of all, the present meta‐analysis shows that, since there is no worsening of urinary symptoms post‐operatively, there cannot be anatomical damage leading to the switching of lower pelvic innervation. This confirms the theory proposed by El‐Toukhy et al. [16], Virtanen et al. [22] and Duru et al. [16, 22, 24], which posits that urinary dysfunction and bladder instability observed in radical hysterectomy are primarily linked to innervation disruption, as highlighted in previous studies [10, 11, 12]. The reduction in endopelvic pressure following hysterectomy, which may decrease pressure on the bladder, is another explanation for these findings. Urinary incontinence can easily occur when the abdominal pressure increases. It is important to consider that the leading cause of hysterectomy in the menopausal population is uterine leiomyomas, a condition that increases endopelvic pressure and may directly contribute to urinary symptoms. This is confirmed by several studies, including El‐Toukhy et al. [16], which demonstrated that the presence of uterine fibroids before surgery was more likely to be associated with an improvement in urinary symptoms after hysterectomy (*p* value < 0.05).

The present meta‐analysis found a reduction in SUI, but the pathophysiology of SUI has not been conclusively established. Therefore, it is essential to emphasise the importance of understanding the pathophysiology to explain these results. In the literature, a distinction is made between intrinsic sphincter insufficiency and hypermobility of the urethra. Intrinsic sphincter insufficiency is thought to involve a reduction in the number of muscle cells or their strength, while hypermobility refers to impaired fixation to the symphysis [25]. Colposuspension and other minimally invasive therapeutic methods aimed at correct a hypermobile urethra by elevating the bladder neck and the proximal part of the urethra behind the pubic symphysis to improve pressure transmission to the urethra [26]. During pelvic floor ultrasound, the mobility of the urethra can be measured in relation to the pubic symphysis and should not be exceed 2 cm during a Valsalva manoeuvre.

Demirci et al., in a study evaluating the bladder neck through perineal ultrasonography of 39 patients before and 1 year after hysterectomy, demonstrated that the position of the bladder neck at rest and under stress did not change from pre‐ to postoperative. They concluded that hysterectomy does not weaken urethral support or increase the rate of SUI [27], as confirmed by the present meta‐analysis. This evidence reinforces the hypothesis that non‐radical hysterectomy does not adversely affect urinary continence mechanisms.

During a hysterectomy, many gynaecologists perform a suspension of the vaginal vault to the uterosacral ligaments as described in the literature. This technique contributes to stabilising pelvic anatomy and may lead to bladder neck elevation, thereby reducing bladder neck mobility and improving continence [28]. In the studies included in this meta‐analysis, only Long et al. [15] describe the suspension of the vaginal dome at the uterosacral ligaments, while the others do not detail this surgical step, representing a limitation of this review.

Furthermore, when comparing the various surgical techniques, there is a statistically significant decrease in urinary frequency for TAH, with an odds ratio (OR) of 0.32 (0.16–0.65), for STH with an OR of 0.53 (0.32–0.88), and for LTH with an OR of 0.34 (0.21–0.56) as well as a decrease in SUI and UUI for TAH with an OR of 0.38 (0.26–0.56) and 0.59 (0.37–0.94), respectively, and a decrease in SUI for LTH with an OR of 0.49 (0.32–0.77).

This finding may be influenced by the higher prevalence of leiomyomas as an indication for surgery, particularly larger uterine size and weight, which were more likely to be managed with the open approach. These data should also be interpreted in light of the publication year of the included studies, many of which predate the widespread adoption of minimally invasive approaches.

### 4.2 Strengths and Limitations

Certain limitations must be considered when evaluating the results of this meta‐analysis. Most of the studies reviewed involved a small number of patients and were not randomised, with only four being randomised. The exclusion of non‐English language studies adds to and may strengthen the inherent selection bias. There were different follow‐up time points, and various cut‐offs considered for increased urinary frequency and nocturia. It is also important to consider the variations in individual perception of urination symptoms. Another key limitation is the influence of age, parity, BMI, menopausal status, the presence of uterine fibroids and a history of diabetes, all of which can influence pre‐ and post‐operative continence [29, 30, 31, 32, 33].

A further limitation concerns the predominance of studies conducted before 2006, when total abdominal hysterectomy was more common and minimally invasive approaches were less prevalent, potentially limiting the generalisability of the results to contemporary surgical practice.

A further limitation is the small number of studies comparing pre‐ and post‐operative urinary parameters, which are essential for objectively assessing urinary incontinence. Additional studies are needed to investigate and extend this aspect. Future research should include larger, randomised studies with standardised methodologies to address these gaps.

### 4.3 Interpretation

The present meta‐analysis demonstrated that simple hysterectomy is associated with a statistically significant reduction in urinary frequency, SUI, UUI and nocturia after surgery. However, adequately powered prospective randomised studies with thorough investigations and symptom assessment using validated questionnaires, along with long‐term follow‐up, are necessary to evaluate the clinical validity of these findings. This would provide a more comprehensive understanding of the effects of simple hysterectomy on urinary symptoms and its underlying mechanisms.

## 5 Conclusion

The present meta‐analysis shows that simple hysterectomy is associated with a statistically significant reduction in urinary frequency, stress urinary incontinence, urge urinary incontinence and nocturia after surgery.

## Author Contributions

R.M.A. and V.D.D. conceived and designed the study. E.D.A., R.M.A. and I.C. performed the literature search and data extraction. A.G., G.B., F.P., C.T. and R.A. contributed to study selection and quality assessment. T.S., G.V., S.R., J.C., M.F. and F.S. provided clinical expertise and contributed to the interpretation of findings. P.S., F.Z., E.D. and G.P. assisted in data verification and manuscript drafting. L.M. supervised the statistical analyses. R.M.A. and V.D.D. drafted the manuscript. All authors critically revised the work for important intellectual content and approved the final version of the manuscript.

## Conflicts of Interest

The authors declare no conflicts of interest.

## Supporting information


**Figure S1:** Flow chart identification process for the study included in the analysis.

**Figure S2:** Forest plot: Changes in the incidence of urinary frequency before and after total hysterectomy, stratified by surgical technique.

**Figure S3:** Forest plot: Changes in the incidence of urinary frequency before and after hysterectomy, stratified by duration of follow‐up (6 and 12 months).

**Figure S4:** Forest plot: Changes in the incidence of stress urinary incontinence before and after total hysterectomy, stratified by surgical technique.

**Figure S5:** Forest plot: Changes in the incidence of stress urinary incontinence before and after hysterectomy, stratified by duration of follow‐up (6 and 12 months and 3 years).

**Figure S6:** Forest plot: Changes in the incidence of urge urinary incontinence before and after total hysterectomy, stratified by surgical technique.

**Figure S7:** Forest plot: Changes in the incidence of urge urinary incontinence before and after hysterectomy, stratified by duration of follow‐up (6 and 12 months and 3 years).

**Figure S8:** Forest plot: Changes in the incidence of nicturia before and after total hysterectomy, stratified by surgical techniques.

**Figure S9:** Forest plot: Changes in the incidence of nicturia before and after hysterectomy, stratified by duration of follow‐up (6 and 12 months).

**Figure S10:** Forest plot: Changes in the incidence of incomplete bladder emptying before and after hysterectomy, stratified by surgical technique.

**Figure S11:** Forest plot: Changes in the incidence of incomplete bladder emptying before and after total hysterectomy, stratified by surgical technique.

**Figure S12:** Forest plot: Changes in the incidence of incomplete bladder emptying before and after hysterectomy, stratified by duration of follow‐up (6 and 12 months).

**Table S1:** Main details of the include articles.

**Table S2:** Risk of bias Robins I tool.

## Data Availability

The data that support the findings of this study are available from the corresponding author upon reasonable request.
