# Supplementary figures and images for: The Impact of Non‐Radical Hysterectomy on Urinary Functions: Evaluation of Symptoms—A Systematic Review and Meta‐Analysis
Source: BJOG. 2025 Oct 17;133(3):391–400. doi: 10.1111/1471-0528.70056 (PMC12770083; doi:10.1111/1471-0528.70056)

# Changes in urinary frequency after total hysterectomy stratify to surgical technique

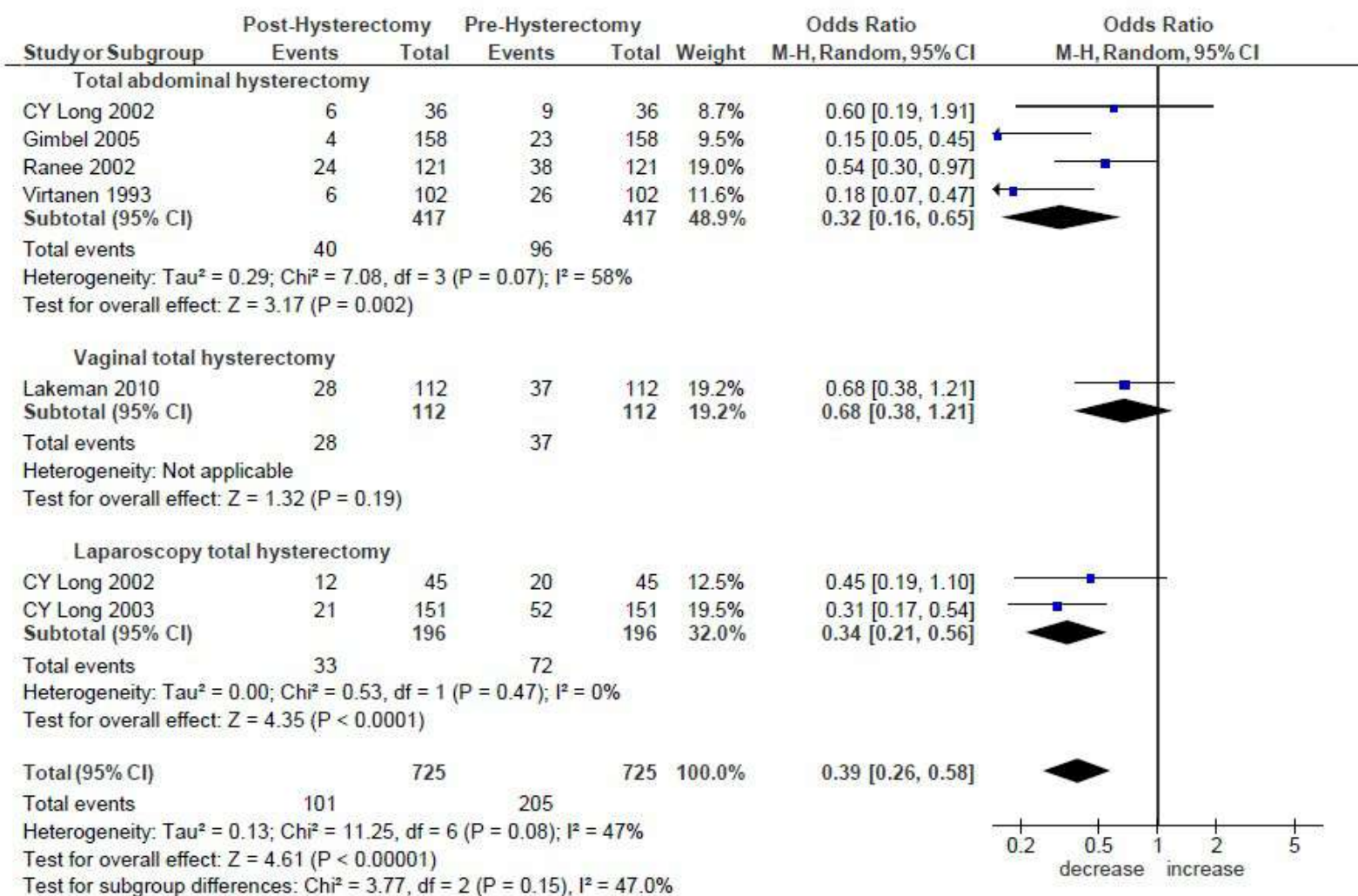

Supplement: Supplementary file 2 — Figure S2: Forest plot: Changes in the incidence of urinary frequency before and after total hysterectomy, stratified by surgical technique. [file BJO-133-391-s006.pdf]

# Changes in nicturia after total hysterectomy stratify to surgical technique

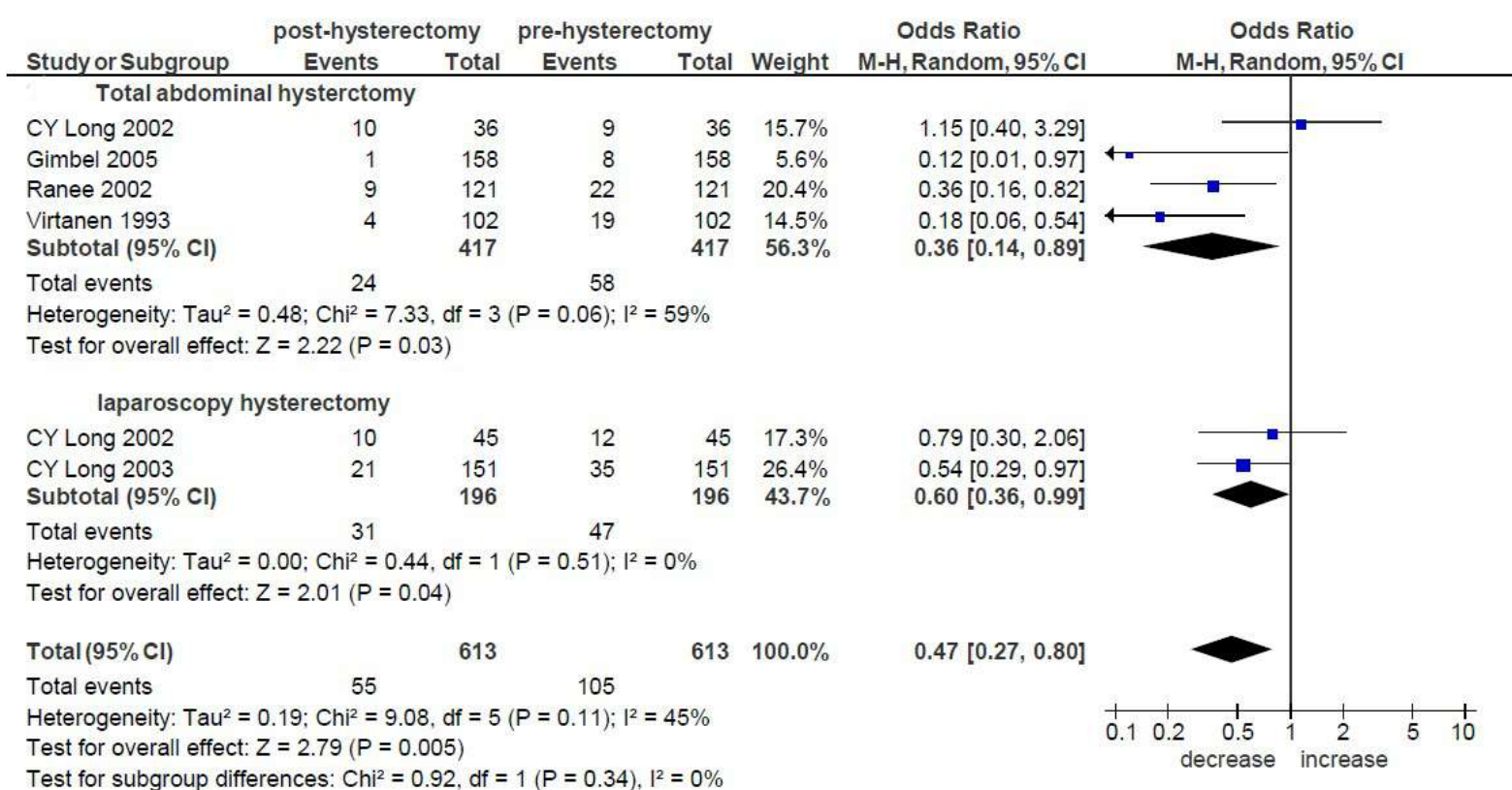

Supplement: Supplementary file 8 — Figure S8: Forest plot: Changes in the incidence of nicturia before and after total hysterectomy, stratified by surgical techniques. [file BJO-133-391-s013.pdf]

# Changes in incomplete bladder emptying stratify to surgical technique

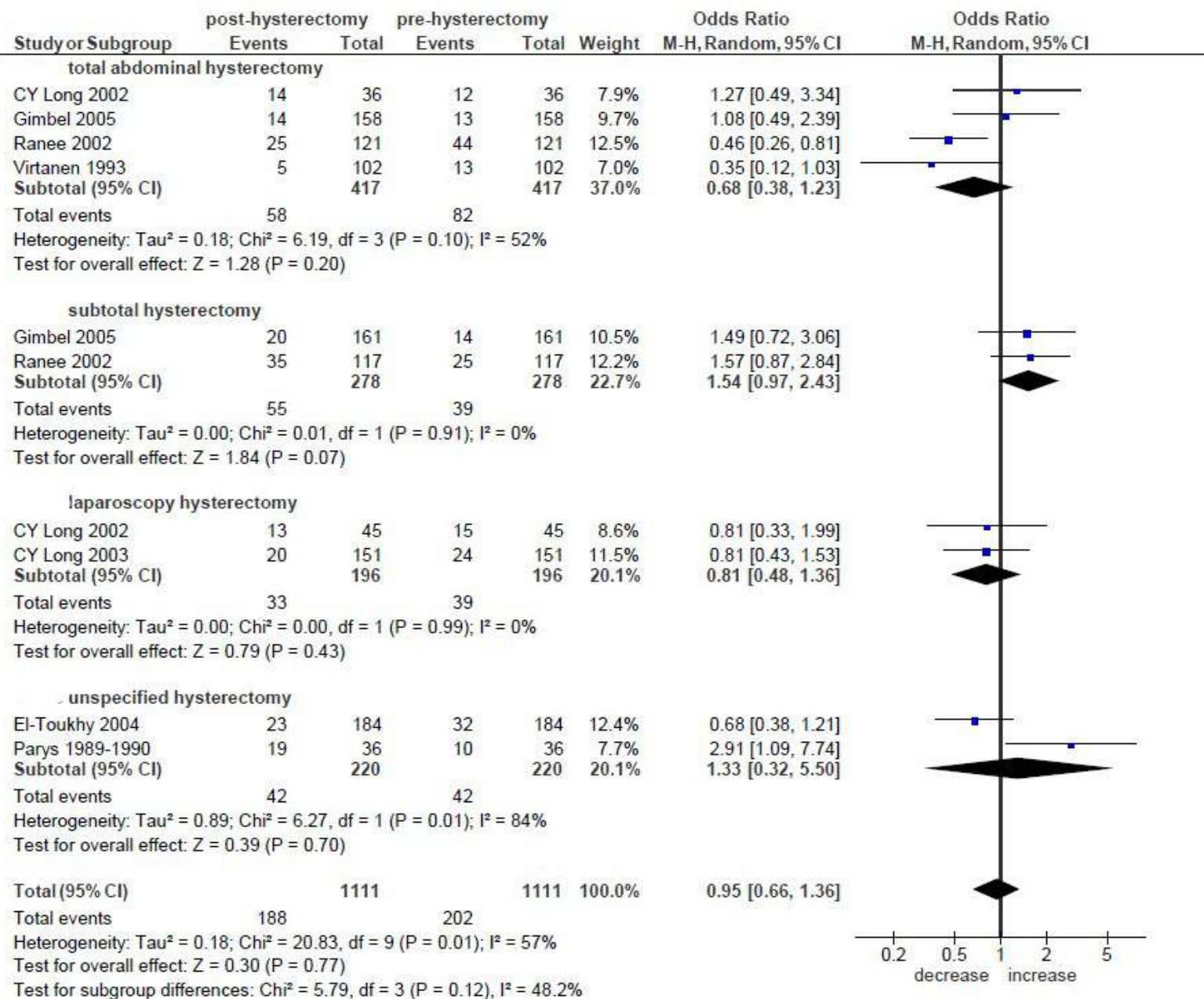

Supplement: Supplementary file 10 — Figure S10: Forest plot: Changes in the incidence of incomplete bladder emptying before and after hysterectomy, stratified by surgical technique. [file BJO-133-391-s003.pdf]

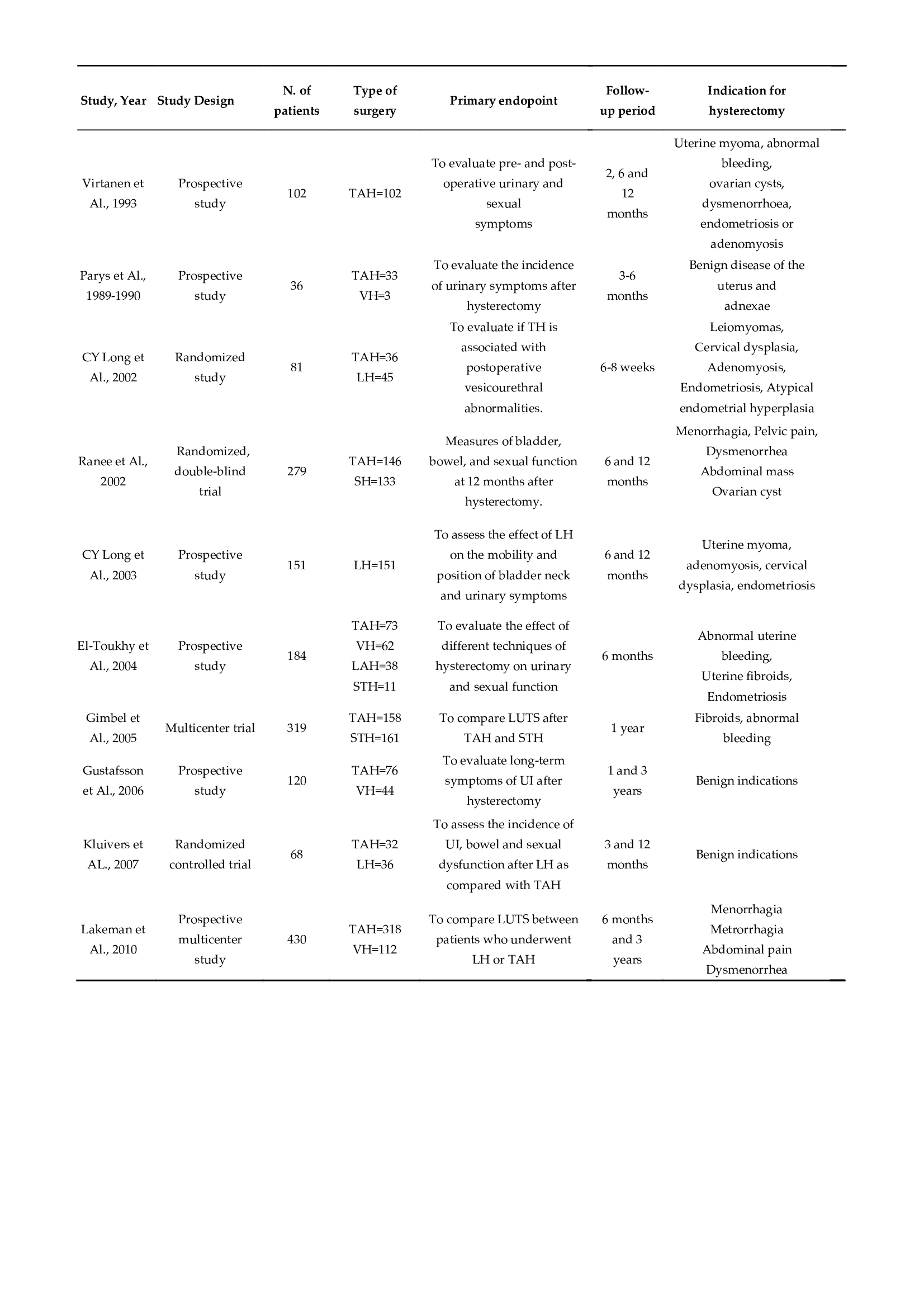

Supplement: Supplementary file 13 — Table S1: Main details of the include articles. [file BJO-133-391-s004.jpg]
