# Supplementary material for: The Impact of Non‐Radical Hysterectomy on Urinary Functions: Evaluation of Symptoms—A Systematic Review and Meta‐Analysis
Source: BJOG. 2025 Oct 17;133(3):391–400. doi: 10.1111/1471-0528.70056 (PMC12770083; doi:10.1111/1471-0528.70056)

Changes in urinary frequency stratify to follow-up (6 months)

Study or Subgroup	post hysterectomy Events	post hysterectomy Total	pre hysterectomy Events	pre hysterectomy Total	Weight	Odds Ratio M-H, Random, 95% CI
Parys 1989-1990	26	36	17	36	11.2%	2.91 [1.09, 7.74]
Virtanen 1993	6	102	26	102	11.7%	0.18 [0.07, 0.47]
CY Long 2003	21	151	52	151	17.3%	0.31 [0.17, 0.54]
El-Toukhy 2004	31	184	49	184	18.4%	0.56 [0.34, 0.93]
Lakeman 2010	88	397	140	430	21.5%	0.59 [0.43, 0.81]
Ranee 2002	48	240	77	240	19.9%	0.53 [0.35, 0.80]
**Total (95% CI)**		**1110**		**1143**	**100.0%**	**0.53 [0.34, 0.83]**
Total events	220		361			

Heterogeneity: $Tau^2 = 0.22$; $Chi^2 = 20.54$, df = 5 (P = 0.0010); $I^2 = 76\%$
Test for overall effect: Z = 2.76 (P = 0.006)

Odds Ratio
M-H, Random, 95% CI

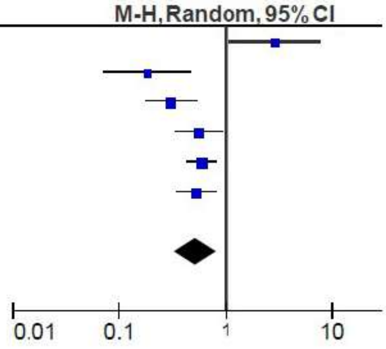

Changes in urinary frequency stratify to follow-up (12 months)

Study or Subgroup	post hysterctomy Events	post hysterctomy Total	pre hysterectomy Events	pre hysterectomy Total	Weight	Odds Ratio M-H, Random, 95% CI
Gimbel 2005	11	319	41	319	33.1%	0.24 [0.12, 0.48]
Ranee 2002	52	240	77	240	40.0%	0.59 [0.39, 0.88]
Virtanen 1993	6	102	26	102	26.9%	0.18 [0.07, 0.47]
**Total (95% CI)**		**661**		**661**	**100.0%**	**0.32 [0.15, 0.68]**
Total events	69		144			

Heterogeneity: $Tau^2 = 0.33$; $Chi^2 = 8.09$, df = 2 (P = 0.02); $I^2 = 75\%$
Test for overall effect: Z = 2.93 (P = 0.003)

Odds Ratio
M-H, Random, 95% CI

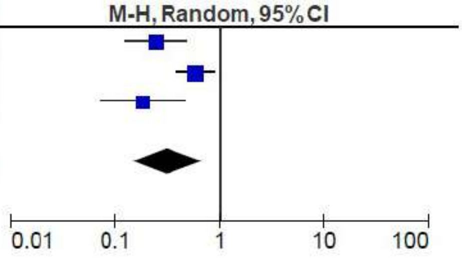

Supplement: Supplementary file 3 — Figure S3: Forest plot: Changes in the incidence of urinary frequency before and after hysterectomy, stratified by duration of follow‐up (6 and 12 months). [file BJO-133-391-s014.pdf]
