# Supplementary material for: The Impact of Non‐Radical Hysterectomy on Urinary Functions: Evaluation of Symptoms—A Systematic Review and Meta‐Analysis
Source: BJOG. 2025 Oct 17;133(3):391–400. doi: 10.1111/1471-0528.70056 (PMC12770083; doi:10.1111/1471-0528.70056)

Changes in stress urinary incontinence after total hysterectomy stratify to surgical technique

	post-hysterectomy		pre-hysterectomy			Odds Ratio	
**Study or Subgroup**	Events	Total	Events	Total	Weight	M-H, Random, 95% CI	Year
**Total abdominal hysterectomy**							
Virtanen 1993	15	102	35	102	12.9%	0.33 [0.17, 0.65]	1993
CY Long 2002	10	36	14	36	7.3%	0.60 [0.22, 1.63]	2002
Gimbel 2005	3	158	15	158	4.9%	0.18 [0.05, 0.65]	2005
Gustafsson 2006	20	68	37	76	12.8%	0.44 [0.22, 0.87]	2006
Kluiver 2007	4	36	9	36	4.7%	0.38 [0.10, 1.35]	2007
**Subtotal (95% CI)**		**400**		**408**	**42.6%**	**0.38 [0.26, 0.56]**	
Total events	52		110				
Heterogeneity: Tau² = 0.00; Chi² = 2.45, df = 4 (P = 0.65); I² = 0%							
Test for overall effect: Z = 4.83 (P < 0.00001)							
**Laparoscopy total hysterectomy**							
CY Long 2002	11	45	18	45	8.5%	0.49 [0.20, 1.20]	2002
CY Long 2003	23	151	44	151	16.5%	0.44 [0.25, 0.77]	2003
Kluiver 2007	7	32	8	32	5.6%	0.84 [0.26, 2.68]	2007
**Subtotal (95% CI)**		**228**		**228**	**30.7%**	**0.49 [0.32, 0.77]**	
Total events	41		70				
Heterogeneity: Tau² = 0.00; Chi² = 0.99, df = 2 (P = 0.61); I² = 0%							
Test for overall effect: Z = 3.13 (P = 0.002)							
**Vaginal total hysterectomy**							
Gustafsson 2006	14	39	17	44	8.7%	0.89 [0.36, 2.17]	2006
Lakeman 2010	49	112	51	112	18.0%	0.93 [0.55, 1.58]	2010
**Subtotal (95% CI)**		**151**		**156**	**26.7%**	**0.92 [0.58, 1.45]**	
Total events	63		68				
Heterogeneity: Tau² = 0.00; Chi² = 0.01, df = 1 (P = 0.93); I² = 0%							
Test for overall effect: Z = 0.36 (P = 0.72)							
**Total (95% CI)**		**779**		**792**	**100.0%**	**0.52 [0.39, 0.70]**	
Total events	156		248				
Heterogeneity: Tau² = 0.05; Chi² = 11.94, df = 9 (P = 0.22); I² = 25%							
Test for overall effect: Z = 4.28 (P < 0.0001)							
Test for subgroup differences: Chi² = 8.49, df = 2 (P = 0.01), I² = 76.4%							

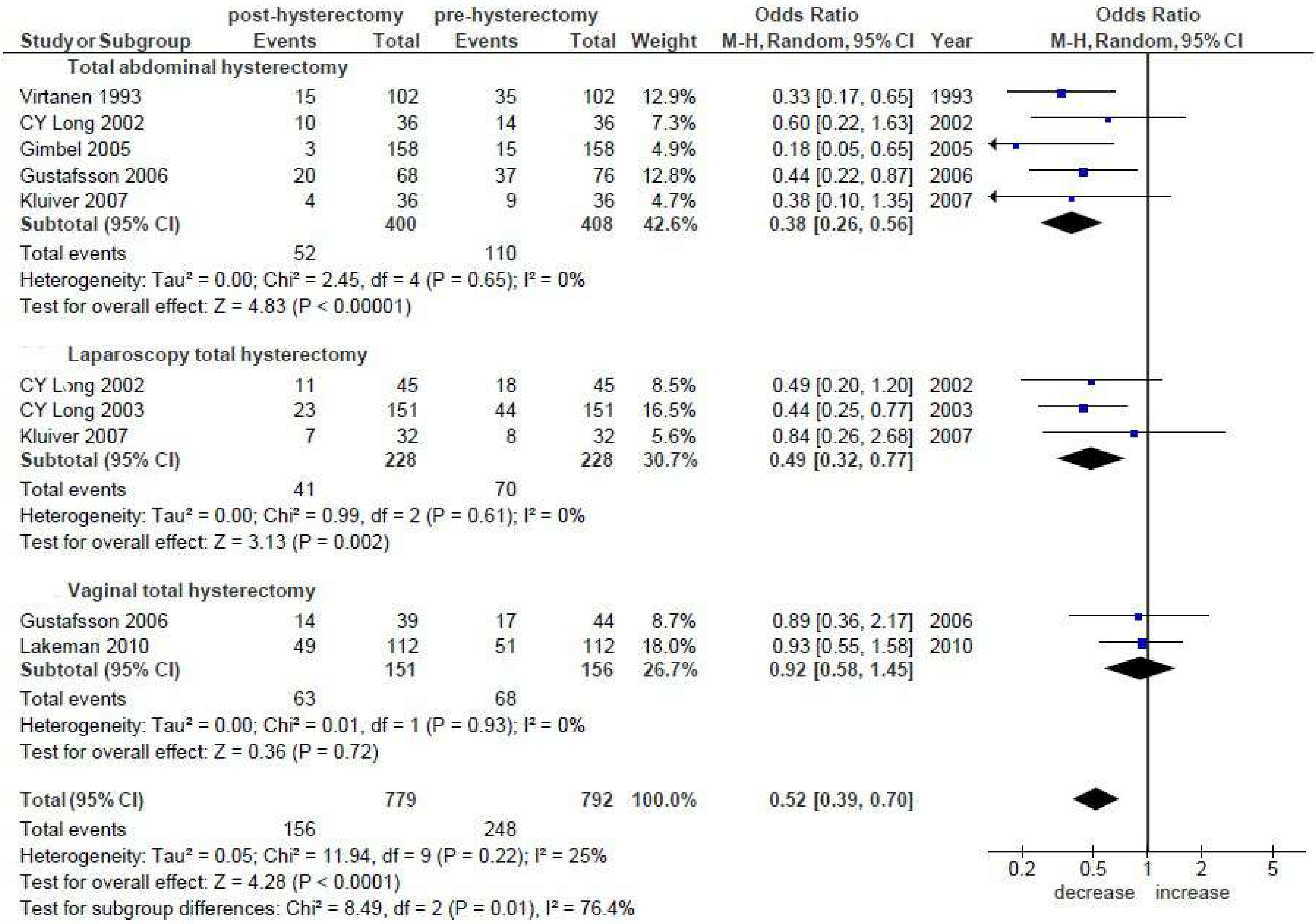

Supplement: Supplementary file 4 — Figure S4: Forest plot: Changes in the incidence of stress urinary incontinence before and after total hysterectomy, stratified by surgical technique. [file BJO-133-391-s011.pdf]
