# Supplementary material for: The Impact of Non‐Radical Hysterectomy on Urinary Functions: Evaluation of Symptoms—A Systematic Review and Meta‐Analysis
Source: BJOG. 2025 Oct 17;133(3):391–400. doi: 10.1111/1471-0528.70056 (PMC12770083; doi:10.1111/1471-0528.70056)

Changes in stress urinary incontinence stratify to follow-up (6 months)

Study or Subgroup	post hysterectomy Events	post hysterectomy Total	pre hysterectomy Events	pre hysterectomy Total	Weight	Odds Ratio M-H, Random, 95% CI	Year
Parys 1989-1990	13	36	9	36	8.7%	1.70 [0.61, 4.68]	1990
Virtanen 1993	15	102	35	102	15.5%	0.33 [0.17, 0.65]	1993
CY Long 2003	23	151	44	151	19.5%	0.44 [0.25, 0.77]	2003
El-Toukhy 2004	35	184	66	184	23.2%	0.42 [0.26, 0.68]	2004
Lakeman 2010	133	397	194	397	33.1%	0.53 [0.40, 0.70]	2010
**Total (95% CI)**		**870**		**870**	**100.0%**	**0.50 [0.35, 0.69]**	
Total events	219		348				

Heterogeneity: $Tau^2 = 0.07$; $Chi^2 = 7.83$, df = 4 (P = 0.10); $I^2 = 49\%$
Test for overall effect: Z = 4.09 (P < 0.0001)

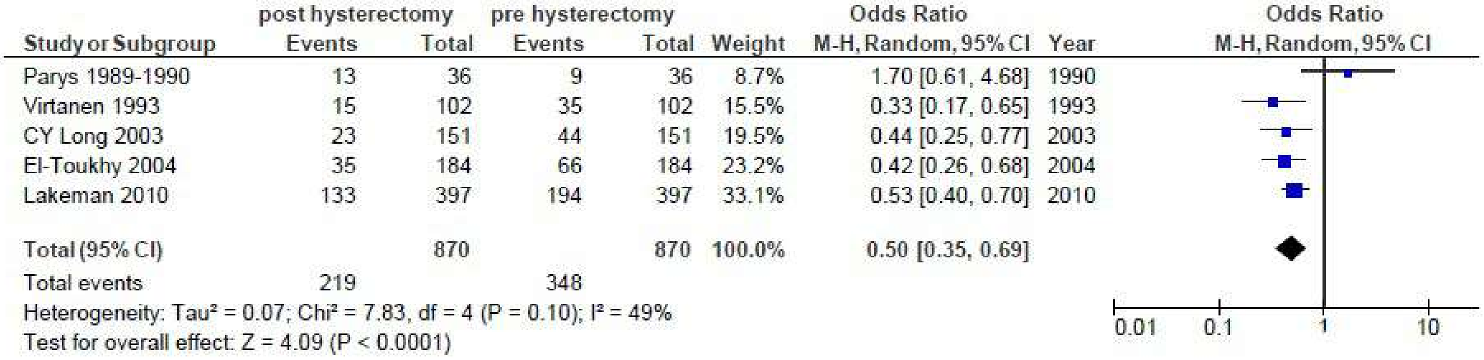

Changes in stress urinary incontinence stratify to follow-up (12 months)

Study or Subgroup	post hysterectomy Events	post hysterectomy Total	pre hysterectomy Events	pre hysterectomy Total	Weight	Odds Ratio M-H, Fixed, 95% CI
Gimbel 2005	11	319	30	319	31.6%	0.34 [0.17, 0.70]
Gustafsson 2006	43	114	54	120	35.8%	0.74 [0.44, 1.25]
Virtanen 1993	15	102	35	102	32.6%	0.33 [0.17, 0.65]
**Total (95% CI)**		**535**		**541**	**100.0%**	**0.48 [0.34, 0.68]**
Total events	69		119			

Heterogeneity: $Chi^2 = 4.64$, df = 2 (P = 0.10); $I^2 = 57\%$
Test for overall effect: Z = 4.06 (P < 0.0001)

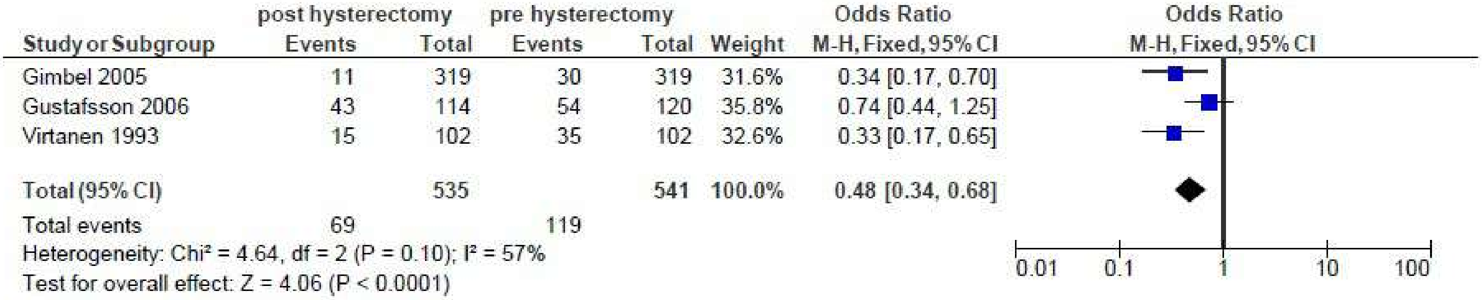

Changes in stress urinary incontinence stratify to follow-up (3 years)

Study or Subgroup	post hysterectomy Events	post hysterectomy Total	pre hysterectomy Events	pre hysterectomy Total	Weight	Odds Ratio M-H, Fixed, 95% CI
Gustafsson 2006	34	107	54	120	26.4%	0.57 [0.33, 0.98]
Lakeman 2010	151	333	194	397	73.6%	0.87 [0.65, 1.16]
**Total (95% CI)**		**440**		**517**	**100.0%**	**0.79 [0.61, 1.02]**
Total events	185		248			

Heterogeneity: $Chi^2 = 1.80$, df = 1 (P = 0.18); $I^2 = 44\%$
Test for overall effect: Z = 1.81 (P = 0.07)

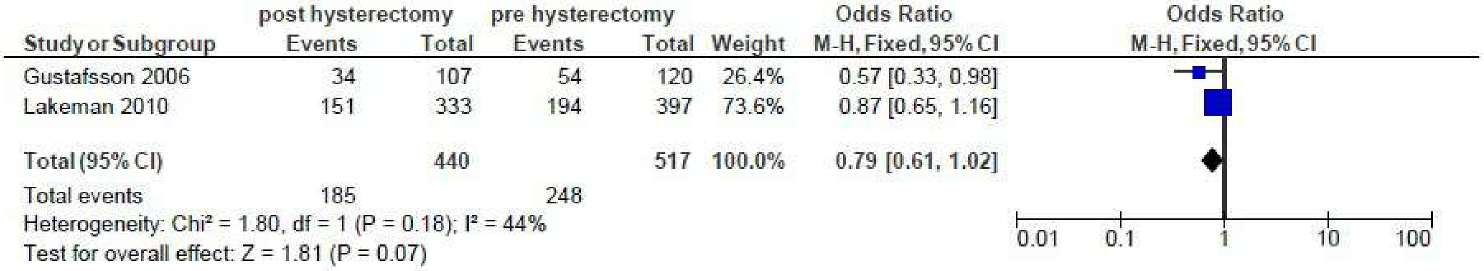

Supplement: Supplementary file 5 — Figure S5: Forest plot: Changes in the incidence of stress urinary incontinence before and after hysterectomy, stratified by duration of follow‐up (6 and 12 months and 3 years). [file BJO-133-391-s002.pdf]
