# Supplementary material for: The Impact of Non‐Radical Hysterectomy on Urinary Functions: Evaluation of Symptoms—A Systematic Review and Meta‐Analysis
Source: BJOG. 2025 Oct 17;133(3):391–400. doi: 10.1111/1471-0528.70056 (PMC12770083; doi:10.1111/1471-0528.70056)

Changes in urge urinary incontinence after total hysterectomy stratify to surgical technique

Study or Subgroup	post-hysterectomy Events	post-hysterectomy Total	pre-hysterectomy Events	pre-hysterectomy Total	Weight	Odds Ratio M-H, Fixed, 95% CI
**Total abdominal hysterectomy**						
CY Long 2002	7	36	8	36	7.4%	0.84 [0.27, 2.64]
Gimbel 2005	2	158	5	158	5.6%	0.39 [0.07, 2.05]
Gustafsson 2006	9	68	14	76	13.1%	0.68 [0.27, 1.68]
Kluiver 2007	0	36	3	36	4.0%	0.13 [0.01, 2.63]
Ranee 2002	14	120	19	120	19.2%	0.70 [0.33, 1.47]
Virtanen 1993	1	102	5	102	5.7%	0.19 [0.02, 1.67]
**Subtotal (95% CI)**		**520**		**528**	**55.0%**	**0.59 [0.37, 0.94]**
Total events	33		54			
Heterogeneity: Chi² = 2.91, df = 5 (P = 0.71); I² = 0%						
Test for overall effect: Z = 2.24 (P = 0.03)						
**Vaginal total hysterectomy**						
Gustafsson 2006	11	39	7	44	5.4%	2.08 [0.71, 6.04]
Lakeman 2010	22	112	22	112	20.2%	1.00 [0.52, 1.93]
**Subtotal (95% CI)**		**151**		**156**	**25.6%**	**1.23 [0.70, 2.14]**
Total events	33		29			
Heterogeneity: Chi² = 1.30, df = 1 (P = 0.25); I² = 23%						
Test for overall effect: Z = 0.72 (P = 0.47)						
**Laparoscopy total hysterectomy**						
CY Long 2002	5	45	6	45	6.1%	0.81 [0.23, 2.88]
CY Long 2003	12	151	11	151	11.6%	1.10 [0.47, 2.57]
Kluiver 2007	0	32	1	32	1.7%	0.32 [0.01, 8.23]
**Subtotal (95% CI)**		**228**		**228**	**19.4%**	**0.94 [0.47, 1.86]**
Total events	17		18			
Heterogeneity: Chi² = 0.60, df = 2 (P = 0.74); I² = 0%						
Test for overall effect: Z = 0.17 (P = 0.86)						
**Total (95% CI)**		**899**		**912**	**100.0%**	**0.82 [0.60, 1.12]**
Total events	83		101			
Heterogeneity: Chi² = 8.30, df = 10 (P = 0.60); I² = 0%						
Test for overall effect: Z = 1.24 (P = 0.22)						
Test for subgroup differences: Chi² = 4.13, df = 2 (P = 0.13), I² = 51.5%						

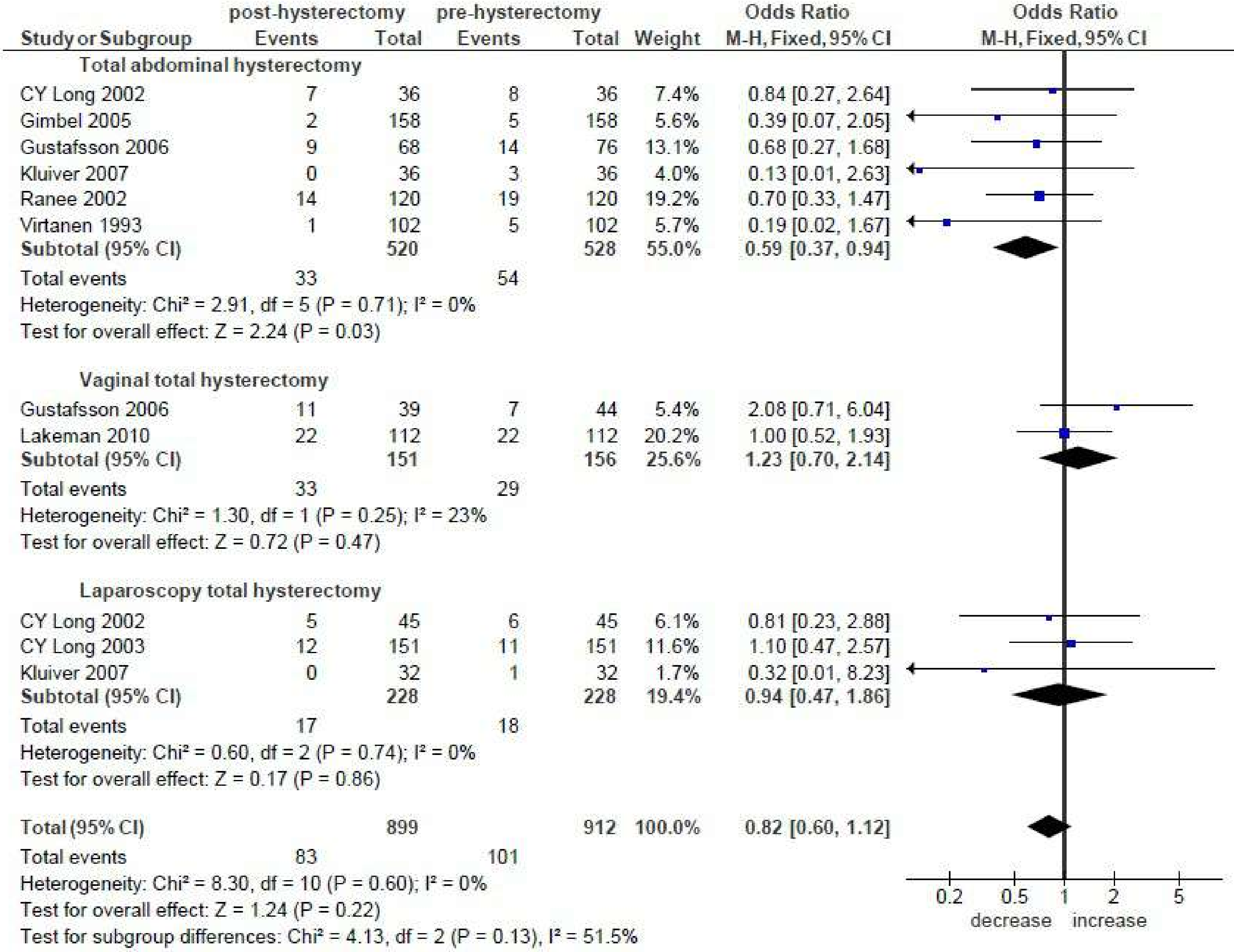

Supplement: Supplementary file 6 — Figure S6: Forest plot: Changes in the incidence of urge urinary incontinence before and after total hysterectomy, stratified by surgical technique. [file BJO-133-391-s005.pdf]
