# Supplementary material for: The Impact of Non‐Radical Hysterectomy on Urinary Functions: Evaluation of Symptoms—A Systematic Review and Meta‐Analysis
Source: BJOG. 2025 Oct 17;133(3):391–400. doi: 10.1111/1471-0528.70056 (PMC12770083; doi:10.1111/1471-0528.70056)

Changes in urge urinary incontinence stratify to follow-up (6 months)

Study or Subgroup	post hysterectomy Events	post hysterectomy Total	pre hysterectomy Events	pre hysterectomy Total	Weight	Odds Ratio M-H, Fixed, 95% CI
CY Long 2003	12	151	11	151	6.4%	1.10 [0.47, 2.57]
El-Toukhy 2004	21	184	32	184	17.8%	0.61 [0.34, 1.11]
Lakeman 2010	59	430	92	430	49.8%	0.58 [0.41, 0.84]
Ranee 2002	26	237	41	237	22.9%	0.59 [0.35, 1.00]
Virtanen 1993	2	102	5	102	3.1%	0.39 [0.07, 2.05]
**Total (95% CI)**		**1104**		**1104**	**100.0%**	**0.62 [0.48, 0.79]**
Total events	120		181			

Heterogeneity: Chi² = 2.18, df = 4 (P = 0.70); I² = 0%
Test for overall effect: Z = 3.79 (P = 0.0001)

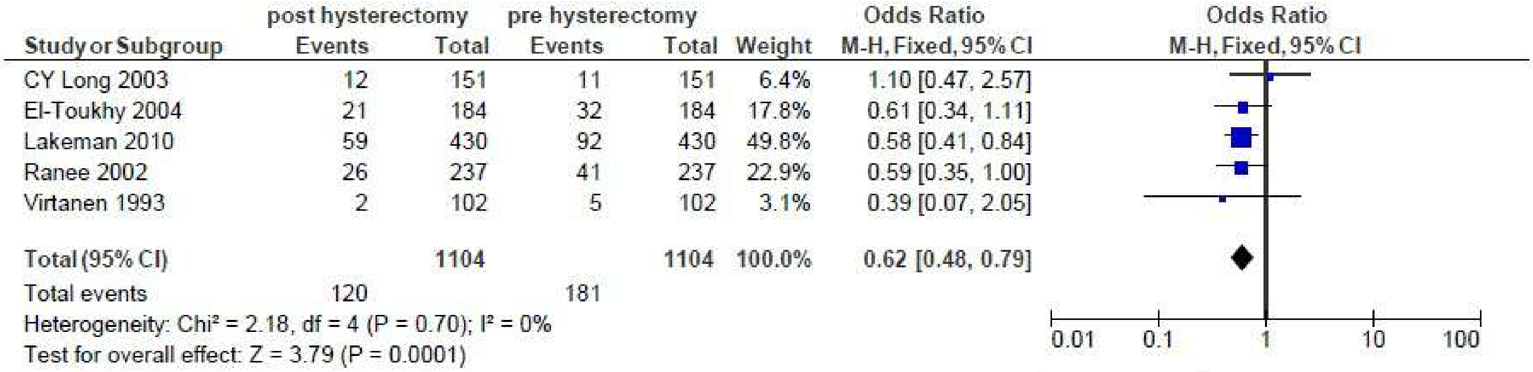

Changes in urge urinary incontinence stratify to follow-up (12 months)

Study or Subgroup	post hysterectomy Events	post hysterectomy Total	pre hysterectomy Events	pre hysterectomy Total	Weight	Odds Ratio M-H, Fixed, 95% CI
Gimbel 2005	9	319	10	319	14.4%	0.90 [0.36, 2.24]
Gustafsson 2006	17	104	21	120	24.2%	0.92 [0.46, 1.86]
Ranee 2002	27	237	41	237	54.0%	0.61 [0.36, 1.04]
Virtanen 1993	1	102	5	102	7.4%	0.19 [0.02, 1.67]
**Total (95% CI)**		**762**		**778**	**100.0%**	**0.70 [0.48, 1.01]**
Total events	54		77			

Heterogeneity: Chi² = 2.48, df = 3 (P = 0.48); I² = 0%
Test for overall effect: Z = 1.89 (P = 0.06)

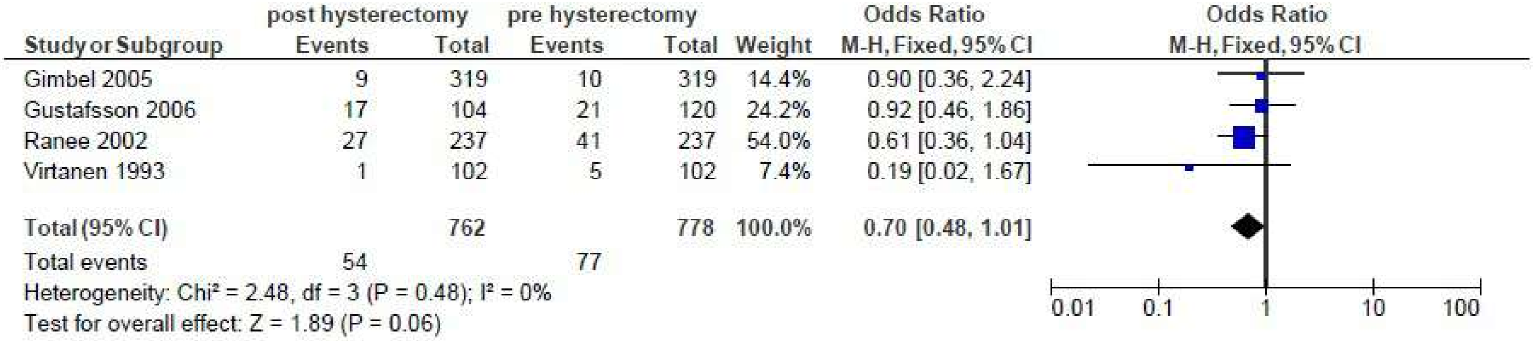

Changes in urge urinary incontinence stratify to follow-up (3 years)

Study or Subgroup	post hysterectomy Events	post hysterectomy Total	pre hysterectomy Events	pre hysterectomy Total	Weight	Odds Ratio M-H, Fixed, 95% CI
Gustafsson 2006	20	107	21	120	20.8%	1.08 [0.55, 2.13]
Lakeman 2010	79	333	92	430	79.2%	1.14 [0.81, 1.61]
**Total (95% CI)**		**440**		**550**	**100.0%**	**1.13 [0.83, 1.53]**
Total events	99		113			

Heterogeneity: Chi² = 0.02, df = 1 (P = 0.89); I² = 0%
Test for overall effect: Z = 0.79 (P = 0.43)

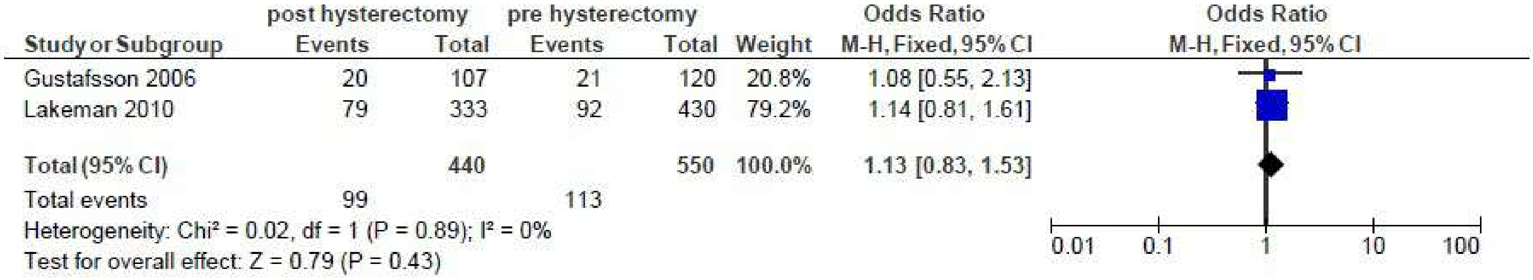

Supplement: Supplementary file 7 — Figure S7: Forest plot: Changes in the incidence of urge urinary incontinence before and after hysterectomy, stratified by duration of follow‐up (6 and 12 months and 3 years). [file BJO-133-391-s010.pdf]
