# Supplementary material for: The Impact of Non‐Radical Hysterectomy on Urinary Functions: Evaluation of Symptoms—A Systematic Review and Meta‐Analysis
Source: BJOG. 2025 Oct 17;133(3):391–400. doi: 10.1111/1471-0528.70056 (PMC12770083; doi:10.1111/1471-0528.70056)

Changes in nicturia stratify to follow-up (6 months)

Study or Subgroup	post hysterectomy Events	post hysterectomy Total	pre hysterectomy Events	pre hysterectomy Total	Weight	Odds Ratio M-H, Fixed, 95% CI
CY Long 2003	21	151	35	151	30.1%	0.54 [0.29, 0.97]
El-Toukhy 2004	9	184	17	184	16.1%	0.51 [0.22, 1.16]
Parys 1989-1990	11	36	7	36	4.9%	1.82 [0.61, 5.41]
Ranee 2002	19	240	33	240	30.3%	0.54 [0.30, 0.98]
Virtanen 1993	2	102	19	102	18.6%	0.09 [0.02, 0.39]
**Total (95% CI)**		**713**		**713**	**100.0%**	**0.51 [0.37, 0.71]**
Total events	62		111			

Heterogeneity: Chi² = 10.73, df = 4 (P = 0.03); I² = 63%
Test for overall effect: Z = 3.95 (P < 0.0001)

Odds Ratio
M-H, Fixed, 95% CI

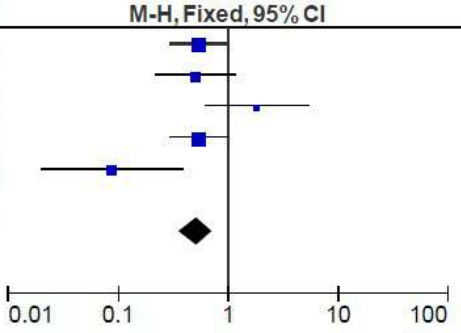

Changes in nicturia stratify to follow-up (12 months)

Study or Subgroup	post hysterectomy Events	post hysterectomy Total	pre hysterectomy Events	pre hysterectomy Total	Weight	Odds Ratio M-H, Fixed, 95% CI
Gimbel 2005	5	319	12	319	26.3%	0.41 [0.14, 1.17]
Ranee 2002	13	240	33	240	69.4%	0.36 [0.18, 0.70]
Virtanen 1993	4	102	2	102	4.3%	2.04 [0.37, 11.40]
**Total (95% CI)**		**661**		**661**	**100.0%**	**0.44 [0.26, 0.75]**
Total events	22		47			

Heterogeneity: Chi² = 3.43, df = 2 (P = 0.18); I² = 42%
Test for overall effect: Z = 3.05 (P = 0.002)

Odds Ratio
M-H, Fixed, 95% CI

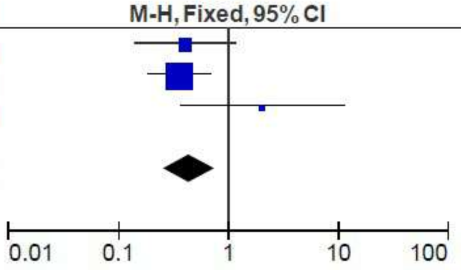

Supplement: Supplementary file 9 — Figure S9: Forest plot: Changes in the incidence of nicturia before and after hysterectomy, stratified by duration of follow‐up (6 and 12 months). [file BJO-133-391-s008.pdf]
