# Supplementary material for: The Impact of Non‐Radical Hysterectomy on Urinary Functions: Evaluation of Symptoms—A Systematic Review and Meta‐Analysis
Source: BJOG. 2025 Oct 17;133(3):391–400. doi: 10.1111/1471-0528.70056 (PMC12770083; doi:10.1111/1471-0528.70056)

Changes in incomplete bladder emptying after total hysterectomy stratify to surgical technique

Study or Subgroup	post-hysterectomy Events	post-hysterectomy Total	pre-hysterectomy Events	pre-hysterectomy Total	Weight	Odds Ratio M-H, Random, 95% CI
total abdominal hysterectomy						
CY Long 2002	14	36	12	36	12.1%	1.27 [0.49, 3.34]
Gimbel 2005	14	158	13	158	16.5%	1.08 [0.49, 2.39]
Ranee 2002	25	121	44	121	25.5%	0.46 [0.26, 0.81]
Virtanen 1993	5	102	13	102	10.1%	0.35 [0.12, 1.03]
**Subtotal (95% CI)**		**417**		**417**	**64.2%**	**0.68 [0.38, 1.23]**
Total events	58		82			
Heterogeneity: Tau² = 0.18; Chi² = 6.19, df = 3 (P = 0.10); I² = 52%						
Test for overall effect: Z = 1.28 (P = 0.20)						
laparoscopy total hysterectomy						
CY Long 2002	13	45	15	45	13.6%	0.81 [0.33, 1.99]
CY Long 2003	20	151	24	151	22.2%	0.81 [0.43, 1.53]
**Subtotal (95% CI)**		**196**		**196**	**35.8%**	**0.81 [0.48, 1.36]**
Total events	33		39			
Heterogeneity: Tau² = 0.00; Chi² = 0.00, df = 1 (P = 0.99); I² = 0%						
Test for overall effect: Z = 0.79 (P = 0.43)						
**Total (95% CI)**		**613**		**613**	**100.0%**	**0.71 [0.49, 1.03]**
Total events	91		121			
Heterogeneity: Tau² = 0.05; Chi² = 6.67, df = 5 (P = 0.25); I² = 25%						
Test for overall effect: Z = 1.80 (P = 0.07)						
Test for subgroup differences: Chi² = 0.19, df = 1 (P = 0.66), I² = 0%						

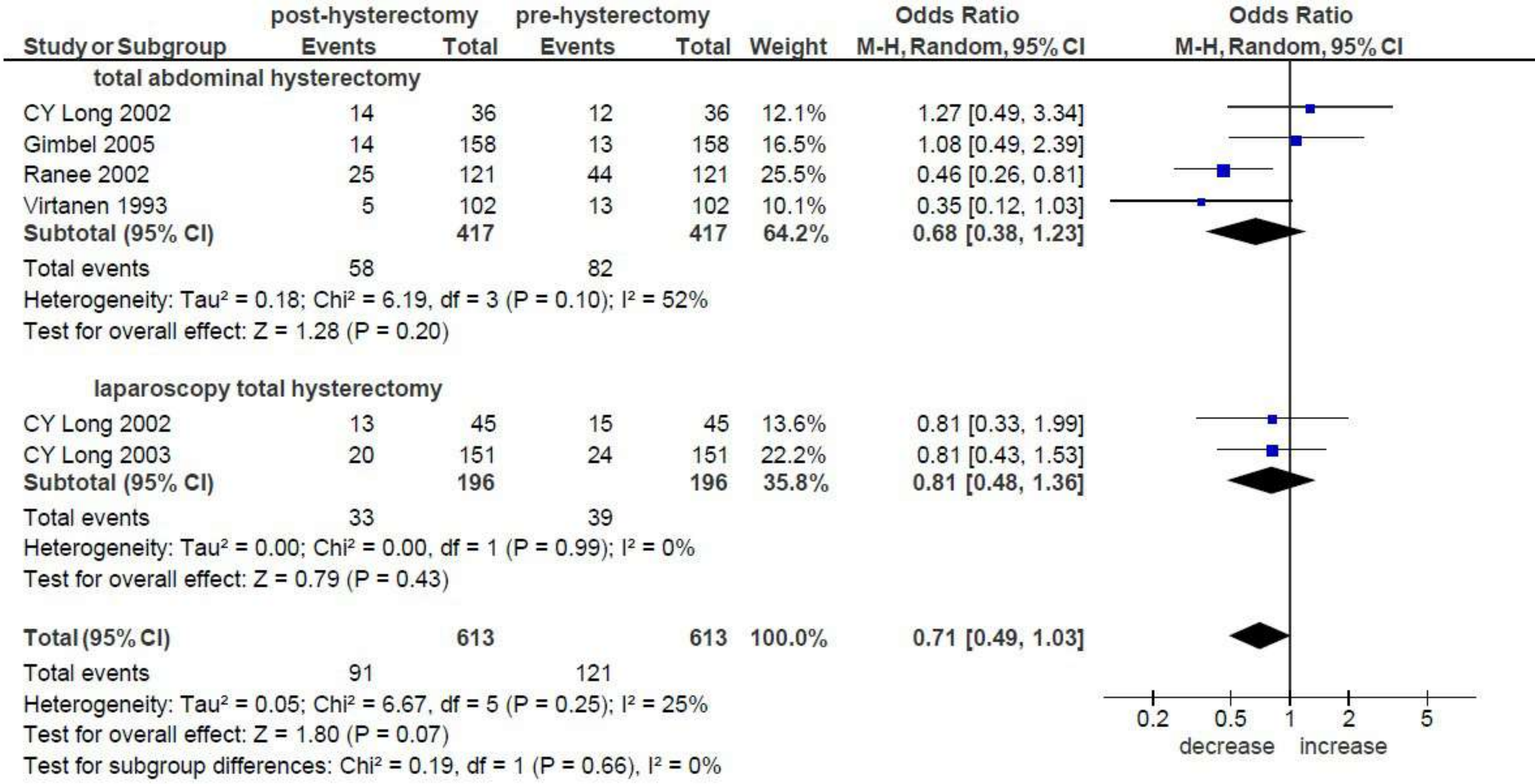

Supplement: Supplementary file 11 — Figure S11: Forest plot: Changes in the incidence of incomplete bladder emptying before and after total hysterectomy, stratified by surgical technique. [file BJO-133-391-s012.pdf]
