# Supplementary material for: The Impact of Non‐Radical Hysterectomy on Urinary Functions: Evaluation of Symptoms—A Systematic Review and Meta‐Analysis
Source: BJOG. 2025 Oct 17;133(3):391–400. doi: 10.1111/1471-0528.70056 (PMC12770083; doi:10.1111/1471-0528.70056)

Changes in incomplete bladder emptying stratify to follow-up (6 months)

Study or Subgroup	post hysterectomy Events	Total	pre hysterectomy Events	Total	Weight	Odds Ratio M-H, Random, 95% CI
El-Toukhy 2004	23	184	32	184	28.8%	0.68 [0.38, 1.21]
Parys 1989-1990	19	36	10	36	20.2%	2.91 [1.09, 7.74]
Ranee 2002	53	238	79	238	32.5%	0.58 [0.38, 0.87]
Virtanen 1993	5	102	13	102	18.5%	0.35 [0.12, 1.03]
**Total (95% CI)**		**560**		**560**	**100.0%**	**0.76 [0.40, 1.47]**
Total events	100		134			

Heterogeneity: $Tau^2 = 0.30$; $Chi^2 = 10.51$, df = 3 (P = 0.01); $I^2 = 71\%$
Test for overall effect: Z = 0.81 (P = 0.42)

Odds Ratio
M-H, Random, 95% CI

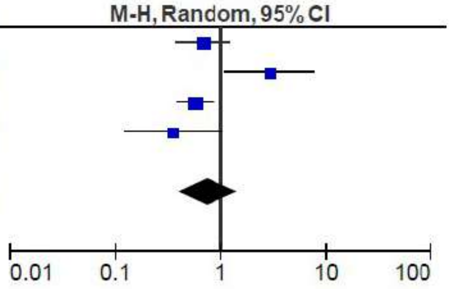

Changes in incomplete bladder emptying stratify to follow-up (12 months)

Study or Subgroup	post hysterectomy Events	Total	pre hysterectomy Events	Total	Weight	Odds Ratio M-H, Fixed, 95% CI
Gimbel 2005	29	319	33	319	27.8%	0.87 [0.51, 1.46]
Ranee 2002	41	238	79	238	60.7%	0.42 [0.27, 0.64]
Virtanen 1993	5	102	13	102	11.5%	0.35 [0.12, 1.03]
**Total (95% CI)**		**659**		**659**	**100.0%**	**0.54 [0.39, 0.73]**
Total events	75		125			

Heterogeneity: $Chi^2 = 5.06$, df = 2 (P = 0.08); $I^2 = 60\%$
Test for overall effect: Z = 3.88 (P = 0.0001)

Odds Ratio
M-H, Fixed, 95% CI

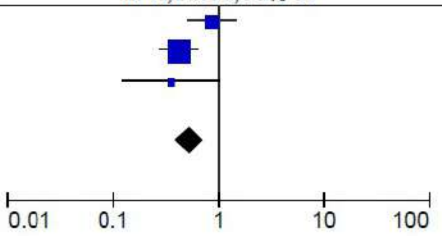

Supplement: Supplementary file 12 — Figure S12: Forest plot: Changes in the incidence of incomplete bladder emptying before and after hysterectomy, stratified by duration of follow‐up (6 and 12 months). [file BJO-133-391-s007.pdf]
