# Supplementary material for: The Impact of Non‐Radical Hysterectomy on Urinary Functions: Evaluation of Symptoms—A Systematic Review and Meta‐Analysis
Source: BJOG. 2025 Oct 17;133(3):391–400. doi: 10.1111/1471-0528.70056 (PMC12770083; doi:10.1111/1471-0528.70056)
Supplement: Supplementary file 14 — Table S2: Risk of bias Robins I tool. [file BJO-133-391-s001.pdf]

Summary Table (ROBINS-I)

Study	Confoundig	Participant Selection	Intervention Classification	Intervention Deviations	Outcome Measurement	Result Selection	Reported Results
Parys et al. (1989, 1990)	High	Moderate	Low	Moderate	Moderate	High	High
Gustafsson et al. (2006)	Low	Low	Low	Low	Low	Low	Low
Lakeman et al. (2010)	Low	Moderate	Low	Moderate	Low	Moderate	Low
Kluivers et al. (2007)	Low	Low	Low	Low	Low	Low	Low
Gimbel et al. (2005)	Low	Low	Low	Low	Low	Low	Low
Cy Long et al. (2002)	Moderate	Low	Low	Low	Low	Low	Moderate
El-Toukhy et al. (2004)	Low	Low	Low	Low	Low	Low	Low
Ranee et al. (2002)	Low	Low	Low	Low	Low	Low	Low
CY Long et. al (2003)	Moderate	Low	Low	Low	Moderate	Moderate	Moderate
Virtanen et al. (1993)	Moderate	Low	Low	Low	Moderate	Low	Moderate
